# New Perspectives of Interferon-alpha2 and Inflammation in Treating Philadelphia-negative Chronic Myeloproliferative Neoplasms

**DOI:** 10.1097/HS9.0000000000000645

**Published:** 2021-11-18

**Authors:** Hans C. Hasselbalch, Richard T. Silver

**Affiliations:** 1 Department of Hematology, Zealand University Hospital, Roskilde, Denmark; 2 Myeloproliferative Neoplasms Center, Division of Hematology and Medical Oncology, Weill Cornell Medicine, New York, New York, USA

In recent years, the use of recombinant interferon-alpha (rIFNα) as the initial treatment of the myeloproliferative neoplasms (MPNs), essential thrombocythemia, polycythemia vera and myelofibrosis, has been increasing. In a subset of patients, treatment with rIFNα for approximately 5 years may result in minimal residual disease (MRD) characterized by hematologic remission, a low *JAK2V617F* allele burden, and normal bone marrow morphology. The important role of chronic inflammation as the driving force for clonal evolution and disease progression and the impact of chronic inflammation upon symptom burden have been substantiated. Here, we highlight timely research questions regarding the use of rIFNα in the future MPN landscape and underscore the importance of early diagnosis and treatment with it to achieve MRD. Based upon the highly encouraging results from combination therapy of stem cell-targeted therapy with rIFNα and the potent anti-inflammatory drug, ruxolitinib, we also place in perspective studies of combinations with older, inexpensive agents (eg, statins, N-acetylcysteine, and colchicine), which have well-established anti-inflammatory and antithrombotic capabilities. Mathematical modeling studies have substantiated the concept that chronic inflammation is a trigger and driver of MPN development, and stress the importance of initiating rIFNα treatment as early as possible. Studies of the impact of rIFNα in individuals carrying the *JAK2V617F* or the *CALR* mutation as clonal hematopoiesis of indeterminate potential (CHIP) are urgently needed to determine whether rIFNα treatment at this early CHIP stage may eradicate the malignant clone. We foresee a bright future for patients with an MPN, in whom early intervention with stem cell-targeted therapy, rIFNα, alone or in combination with drugs targeting the chronic inflammatory state, may allow many to achieve MRD, thus becoming candidates for clinical trials employing vaccines leading to the possibility of cure.

## Interferon-alpha2 in the myeloproliferative neoplasms

In 1985, Linkesch et al reported for the first time that rIFNα controlled myeloproliferation in patients with an MPN accompanied with severe thrombocytosis.^1,2^ A few years later, Silver^3^ demonstrated the safety and efficacy of rIFNα treatment in patients with polycythemia vera (PV) and afterward, its value in the proliferative phase of myelofibrosis (MF) was reported,^4^ resulting in normalization of marrow architecture and cellularity, and reduction in degree of fibrosis to normal.^5^ Many subsequent studies in more than a thousand patients have confirmed that rIFNα is safe and effective for treating essential thrombocythemia (ET), PV, and early-stage MF patients: in ET, it normalizes elevated platelet counts within weeks to months in the large majority of patients; in PV, it reduces or eliminates the phlebotomy requirement and the degree of pruritus, normalizes elevated leukocyte and platelet counts and reduces spleen size; in MF patients, it reduces or normalizes elevated leukocyte- and platelet counts and—as noted above—may also induce regression of bone marrow fibrosis in some patients after long-term treatment.^5^ All these studies have been thoroughly described in several recent reviews.^6–14^ In a single-arm study of 55 patients with PV, rIFNα therapy resulted in significant reduction in need for phlebotomy and in thrombotic events.^15^ In the largest retrospective study of 470 PV patients from the same institution, improved myelofibrosis-free survival and probably overall survival were observed in rIFNα-treated patients compared to those treated with hydroxyurea (HU) or phlebotomy only (PHL-O).^16^

Recent studies have elucidated novel mechanisms of action of rIFNα therapy in the MPNs, which basically and simplistically depends on physiological stem cell exhaustion and/or depletion. In MPN mice, rIFNα can directly eliminate malignant disease-initiating cells by inducing changes in the cell cycle and apoptosis.^17–19^ Tong et al, by single-cell transcriptomic profiling coupled with mutation detection, showed that in patients with ET, *JAK2V617F* megakaryocytic stem cells had elevated interferon signaling. Upon treatment, homozygous mutant HSCs had a quiescent signature in comparison to heterozygous stem cells, which underwent enhanced apoptosis.^20^

The interest in using rIFNα long-term was abetted by the reports of it decreasing the *JAK2V617F* allele burden in PV.^21–26^ MRD, noted in a subset of patients, was defined as clinical and hematologic remission, a *JAK2V617F* allelic burden <1% and normalization of marrow morphology.^23,24,27^ These results could be sustained after discontinuation of rIFNα for more than 2–3 years.^23,24,27^ The long-term impact of rIFNα in patients following discontinuation of therapy may reflect rIFNα reprogramming defective immune cells and restoring competent “tumor immune surveillance.”^13,28–30^

Despite these impressive results, these were primarily based upon phase 2 or single-arm studies and did not satisfy regulatory requirements._11,31_ Accordingly, rIFNα was used off label and in the United States, required tedious insurance company approval prior to its use. This has recently changed in Europe because of the licensing of ropeg-rIFNα-2b (Besremi) for the treatment of European LeukemiaNet (ELN) defined high-risk PV patients without symptomatic splenomegaly. The safety and efficacy of this novel drug characterized by a proline pegylated bond have been demonstrated in several studies; it has the advantage of administration every second or third week.^32–35^ Its toxicity profile may be less than with either pegylated rIFNα-2a (Pegasys) or pegylated rIFNα-2b (PegIntron). However, there have been no comparative trials to verify this presumption.

## The future interferon-MPN landscape

In the future, several research questions regarding the use of rIFNα will hopefully be addressed:

### How does chronic inflammation, caused by smoking, impact the response to rIFNα?

Smoking elicits a massive systemic inflammatory stimulus, causing leukocytosis and, sometimes, thrombocytosis.^36^ The JAK-STAT and NF-kappaB signaling pathways are activated in both smokers and in patients with MPNs. Both share elevated levels of several pro-inflammatory cytokines, in vivo activation of leukocytes and platelets, endothelial cell dysfunction, and increased systemic oxidative stress.^36^ In this context, it has been suggested that smoking may trigger MPN development and may also enhance clonal evolution as a consequence of inflammation-mediated genomic instability.^36^ Indeed, the concept of smoking as a risk factor for the development of an MPN has been substantiated in recent studies.^37–40^ Since smoking may be a likely trigger and driver of clonal evolution in patients with an MPN and since smoking, per se, gives rise to erythrocytosis, leukocytosis, and sometimes thrombocytosis,^37^ it increases the thrombotic risk associated with an MPN. A recent study has shown that smoking impairs molecular response and reduces overall survival in MPN patients treated with rIFNα.^41^

### What are the reasons for rIFNα resistance or intolerance in the MPNs?

In some patients rIFNα may elicit a sustained “inflammatory syndrome,” characterized by fatigue and muscle and joint pain, necessitating its dose reduction, thereby perhaps leading to its discontinuation because of diminishing efficacy. Currently, it is unknown which mechanisms are responsible for the emergence of this “inflammatory syndrome,” but several may be operative. First, our clinical experience indicates that patients with advanced MPN-disease and a large tumor burden, for example, patients with myelofibrosis and massive splenomegaly, do not tolerate rIFNα well, owing to its side effects. Perhaps, this intolerance might be explained by a rIFNα-induced cytokine storm. This increase may be temporary and may decline in concert with rIFNα-mediated reduction in tumor burden. In this time-frame, adding a potent anti-inflammatory drug (eg, ruxolitinib or prednisolone) might be a rational approach as addressed below. Second, studies are ongoing to explore whether such autoimmune and inflammatory side effects may be associated with a particular human leukocyte antigen (HLA) tissue type. In this regard, it is worth considering whether MPN patients intolerant to rIFNα may have a predisposition for developing autoimmunity which then is elicited or exacerbated during treatment with rIFNα. There are reports that patients with *TET2*-mutations have impaired response to treatment with rIFNα.^42–45^ Recently, Stetka et al^46^ demonstrated that genetic loss of *DNMT3A* conferred resistance to treatment with rIFNα in a *JAK2V617F* driven MPN mouse model. An association between *DNMT3A*-mutations and impaired response to rIFNα is supported by the Danish DALIAH-trial, in which *DNMT3A*-mutations emerged on treatment more frequently than non-*DNMT3A*-mutations among patients not achieving complete hematological remission (CHR).^47^ Third, as alluded to previously, inflammatory signaling is associated with a diminished effect of rIFNα.^48^ All rIFNα effects are elicited through interaction with type I IFNα receptors, the IFNα-2AR1 and IFNα-2AR2 chains. Inflammation-mediated downregulation of IFNα-2AR1 is associated with refractoriness to rIFNα.^49^ Noteworthy in this context is that the inflammatory cytokines interleukin 1-alpha (IL-1-α) and tumor necrosis factor alpha (TNF-α) stimulate IFNα-2AR1 degradation and accordingly attenuate IFNα-2a signaling.^48^ Similarly, unresponsiveness to rIFNα-2a in hepatitis patients may be explained by oxidative stress, also impairing IFNα-2a signaling.^50^ MPNs are associated with increased levels of several inflammatory cytokines, including IL1-α and TNF-α, the highest levels have been reported in patients with advanced myelofibrosis.^51^ Thus, treating patients with rIFNα at the earliest disease stage possible, when inflammation is less pronounced, seems a more rational approach rather than a “watch and wait policy,” which permits the malignant clone to expand, thus increasing its inflammatory load.^13^ The early intervention with rIFNα has recently been supported by mathematical modeling studies. These show that the earlier rIFNα is started in PV and related neoplasms, the more rapid the decline in the *JAK2V617F* allele burden. This results in a shorter treatment period in order to obtain a major molecular remission.^52^ Early rIFNα treatment of patients with primary and secondary myelofibrosis may result in regression of bone marrow fibrosis and improved marrow architecture and cellularity.^44,53^ Recently, germ-line genetic factors have been shown to influence rIFNα-response in patients with PV, which may affect rIFNα resistance or intolerance.^54,55^

### How does rIFNα-2a impact the chronic inflammatory state and defective tumor immune surveillance in the MPNs?

By normalizing elevated leukocyte and platelet counts, rIFNα helps minimize the sustained release of inflammatory cytokines and chemokines and concurrently improves immune cell function which is important for intact tumor immune surveillance.^56–58^ Patients with MPNs are subject to an increased risk of second cancers,^59–63^ which have an inherently worse prognosis compared to the same cancer as in an MPN-naive person.^60^ Thrombocytosis is a worse prognostic factor in several cancers, and platelets enhance cancer invasiveness and metastatic potential.^64^ Thus, leukocytosis and thrombocytosis in patients with MPNs may contribute to the increased risk of second cancers and inferior survival, both by eliciting defective tumor immune surveillance and by increasing cancer invasiveness.^60,63,64^ rIFNα may restore normal tumor surveillance by increasing the number of several types of immune cells, including dendritic cells, T-cells and natural killer (NK)-cells.^13,28,30^ In addition, rIFNα upregulates previously downregulated HLA-genes, thereby improving tumor cell killing.^65,66^ Furthermore, rIFNα also downregulates or normoregulates *JAK2V617F*-induced expression of the immune check point programmed-cell-death-ligand 1 (PD-L1),^67^ thereby impairing PD-L1 mediated immune escape.^68^ Whole blood gene expression studies indicate that rIFNα treatment decreases expression of genes involved in regulation of inflammation and enhances expression of genes of importance for immune cell function.^69^ Whole blood transcriptional profiling studies have also shown that rIFNα has a major impact upon deregulated oxidative stress genes and antioxidative defense genes.^70^ Importantly, downregulation of several upregulated thromboinflammatory genes, including the PADI4 gene has been demonstrated. This gene is required for neutrophil extracellular trap (NET) formation and thrombosis development.^71^

### Interferon-alpha2 combination therapies: combination with ruxolitinib

In PV, rIFNα-2a monotherapy, together with targeted therapeutic phlebotomy, normalizes elevated blood cell counts within a few months, often accompanied by a decrease in the *JAK2V617F* allele burden.^21–26,32–35,43^ However, major molecular remissions are rare within the first 2 years of therapy and a minority of patients with PV may require a few phlebotomies per year despite 2–3 years of treatment. We prefer to gradually increase the dose of rIFNα, starting with a low dose of pegIFNα-2a 45 µg/week; if no normalization of peripheral cell counts after 1–2 months, we increase the dose to 90 µg/week. Rarely, patients need 135 or 180 µg/week. About 15%–40% of patients do not tolerate rIFNα because of symptoms of toxicity, usually because the doses used have been too high.^16,21,22,25^ However, even with low-dose pegIFNα-2a, 45 µg/week, the discontinuation rate in the DALIAH-trial reached 50%.^72^ Since intolerance may be partly explained by rIFNα-exacerbated inflammation, combination therapy of rIFNα with an anti-inflammatory drug such as ruxolitinib may dampen inflammation and restore its sensitivity and enhance efficacy.^73,74^ Taking into account that ruxolitinib inhibits canonical type 1 IFN-signaling through JAK1 inhibition, such a combination therapy might theoretically have antagonistic effects. However, our clinical trials in PV and MF patients who had been previously intolerant or refractory to rIFNα-2a monotherapy have shown this combination therapy to be both safe and effective.^75–77^ These highly interesting and encouraging findings may be explained by several mechanisms, including the fact that ruxolitinib has a half-time of only a few hours leaving an open window of several hours per day for IFN-signaling. Other mechanisms might be that JAK/STAT inhibition dampens inflammation, which has been reported to impair IFN-signaling by degradation of the IFN-receptor as alluded to above.^48,49^ The rationale for this combination has been substantiated by in vivo murine studies of *JAK2V617F* hematopoietic stem cells, demonstrating distinct effects of ruxolitinib and rIFNα.^19^ However, the results require validation in both newly diagnosed PV and MF patients.^14,75–78^

Since statins may enhance the efficacy of ruxolitinib^79^ and rIFNα,^80^ triple therapy of rIFNα + ruxolitinib + statin may be a highly effective triplet, but obviously requires evaluation in future trials.^13,74^ A recent study indicates hypoxia-inducible factor 1 (HIF-1) as a new therapeutic target in *JAK2V617F*-positive MPNs, demonstrating the potential of the peptide antibiotic, echinomycin, alone and in combination with ruxolitinib, to selectively target *JAK2V617F*-positive cells inducing apoptosis and cell cycle arrest.^81^ In this context, it may be interesting to combine a HIF-1-inhibitor and JAK1-2 inhibitor with rIFNα, which might further enhance the synergistic effects of combining ruxolitinib and rIFNα.

### Combination with statins

Statins have been suggested as potentially useful enhancers of rIFNα in treating MPNs, owing to their antiproliferative, antiangiogenic, proapoptotic, and anti-inflammatory attributes.^82,83^ A recent study showed that PV patients who are treated with statins require fewer phlebotomies than those who are not.^84^ Although the underlying mechanisms are elusive, a statin-induced lowering of inflammation in JAK-STAT signaling is a possible explanation.^82,83^ In this context, we note that low-density lipoproteins (LDLs) amplify cytokine-signaling in chronic lymphocytic leukemia cells.^85^ Thus, future studies should address whether LDLs enhance proliferation of MPN cells in response to inflammatory signals. Because patients with MPNs have an increased risk of second cancers^59–63^ and because statins have been shown to reduce cancer-associated mortality by 15%,^86^ their role in the treatment of MPNs is currently under investigation.

### Combination with HU

HU is the drug most often used in the treatment of patients with MPNs. However, concern has been raised regarding its leukemogenic potential for treatment exceeding 10–15 years.^87^ Therefore, physicians at many MPN centers are cautious about using HU in patients <60 years. Theoretically, combination therapy of rIFNα with HU might nevertheless be a relevant approach. By inducing so-called immunogenic cell death, HU may expose tumor antigens to the immune system. Studies have shown that HU upregulates the immunoreceptor, natural-killer group 2, member D (NKG2D), originally identified in NK cells.^88^ This immunoreceptor recognizes ligands that are upregulated on tumor cells. Accordingly, HU may enhance the susceptibility of clonal MPN cells to NK-mediated cytolysis.^88^ Since rIFNα both upregulates NKG2D^89^ and increases NK-cell cytotoxic activity, the combination of rIFNα and HU might exert a synergistic immune killing effect on the malignant clone in excess of their direct cell killing effects. HU potently lowers elevated levels of inflammatory cytokines in patients with sickle cell anemia (SCA), thereby decreasing the inflammatory state and reducing the risk of thrombosis.^90^ Although the impact of HU upon increased inflammatory cytokines has not been studied systematically in patients with MPNs, HU could reduce cytokines in MPN patients, and enhance the efficacy of rIFNα, dampened by concurrent inflammation. HU might also alleviate the inflammation-mediated flu-like symptoms elicited by rIFNα. Preliminary data indicate that fluctuating cell counts during treatment of PV with HU may contribute to an increased thrombotic risk within the first 3–6 months after starting the drug.^91^ Since rIFNα causes normalization of elevated cell counts without such oscillations, a combination of both drugs during the first months after diagnosis might offer less toxicity than single drug treatment and perhaps reduce further the increased risk of thrombosis.^91^

### Combination with vaccination and immune checkpoint inhibitor strategies

Recently, the CALR and the *JAK2V617F* mutations, present in >90% of MPN patients, have been shown to be immunogenic neo-antigens.^92–95^ Importantly, the immune responses in *JAK2V617F*-positive patients are minor compared to those of *CALR*-positive patients. This small discrepancy may be related to the single amino acid difference between the mutant *JAK2V617F* epitope and the wild type JAK2 epitope, whereas the mutant *CALR* C-terminus spans 36 amino acids.^94^ Furthermore, patients with MPN display frequent and strong T-cell responses against the PD-L1 and arginase-1.^96,97^ Thus, peptide vaccination with either *JAK2* mutant or *CALR* mutant epitopes in combination with vaccination against PD-L1 and/or arginase may be a new and potentially curable treatment modality for MPN patients.^98^ This requires pretreatment with rIFNα, either as monotherapy or in combination with ruxolitinib, to achieve MRD, a prerequisite for eliminating the residual clone by vaccination strategies.^99^ Studies of the safety and efficacy of immune checkpoint inhibitors, for example, blocking PD-L1, are currently under investigation in patients with myelofibrosis.^100^ PD-L1 is upregulated on *JAK2V617F* mutated cells,^67,68^ prohibiting a tumor-specific immune response against the malignant *JAK2V617F*-mutated cells by binding to tumor-specific T cells, resulting in their inactivation.^68^ The *JAK2V617F* mutation also generates reactive oxygen species,^101,102^ which inhibit T-cell function.^103^ Accordingly, there are several rationales for including rIFNα in future studies of vaccine and immune checkpoint inhibitors. rIFNα would enhance the tumor-specific immune responses by boosting immune cell function and lowering the *JAK2V617F* allelic burden resulting in a decreased generation of reactive oxygen species, which in turn impairs T-cell function, as mentioned above.^103^

## Discussion

The impact of chronic inflammation as an important driving force for clonal expansion and evolution in patients with MPNs opens a new horizon for combination studies. Such studies preferentially should include rIFNα, which is the only disease-modifying drug that can induce deep molecular remission and normalization of marrow morphology in a subset of patients. We believe these beneficial effects are likely attributed to the stem-cell targeting potential of rIFNα which boosts virtually all immune cells engaged in “tumor immune surveillance.” The encouraging results of combining rIFNα with ruxolitinib^73–78^ may introduce combination studies with currently available and inexpensive drugs, such as statins, and N-acetylcysteine, which all have shown potent anti-inflammatory, antithrombotic, and anticancer capabilities.^82–84,104^ The intriguing combination of rIFNα and arsenic may have the potential to eradicate the *JAK2V617F* clone.^105^ Since HU does not induce sustained normalization of elevated cell counts in PV patients, it may be rational to combine lower doses of HU with rIFNα, thereby reducing the increased thrombotic risk in PV and reducing rIFNα toxicity. Mathematical modeling studies have shown that the earlier treatment with rIFNα is instituted the more likely the chance of obtaining rapid and deep molecular responses.^52^

It would be interesting to study the impact of rIFNα treatment in the CHIP phase to determine whether inhibiting *JAK2V617F* would also inhibit prodromal thrombotic events and overt MPN disease development. Similarly, studies of the impact of IL-1b or IL-6R blockade upon the kinetics of the *JAK2V617F* mutation in the CHIP phase might unravel the important role of chronic inflammation for abetting clonal expansion. Future research should also focus on the use of colchicine. This old and inexpensive drug has recently been shown to decrease the risk of cardiovascular events,^106^ likely owing to its impact upon circulating inflammatory cytokines, the inflammasome, and subsequently NETosis generation.^107^ Studies on the impact of colchicine on the kinetics of the driver mutations, *JAK2V617F* and *CALR*, and blood cell counts both in the CHIP stage and in MPN patients are urgently needed.

In conclusion, MPNs are not truly orphan diseases because they are frequently underdiagnosed.^108^ MPNs carry an inherent early and increased risk of life-threatening thrombotic events^109,110^ and an increased risk of second cancers,^59–63^ underscoring the urgent need for their earlier detection. Fortunately, at last, our early intervention concept with rIFNα,^9,11,13–16,23,24,27^ now routinely used at several MPN centers, has recently been substantiated, irrespective of conventional risk-stratification schema.^47^ The randomized trial of ropeg-interferon-α2b in early-stage ELN high-risk PV patients also supports this concept,^35^ as does the treatment of ELN low-risk patients.^111,112^ Pegylated rIFNα is also an effective therapy for patients with PV (or ET) previously refractory and/or intolerant of HU.^15,113^ Importantly, as previously discussed a recent study of 470 PV patients has shown that rIFNα yields improved myelofibrosis-free and overall survival,^16^ as does a recent meta-analysis.^114^ These data, together with those generated from a large number of single-arm studies which enrolled more than 1,000 patients over the past 30 years,^15,16,35,114–116^ will result in more MPN patients who will be fortunately treated with rIFNα in the future.

## Acknowledgments

We thank Ms. Mara Sanderson for assistance in the preparation of this manuscript.

## Disclosures

RTS: Chair, Data Safety Monitoring Board (DSMB), PharmaEssentia Corp.; Consultant, AbbVie. HH: Research Grant, Novartis A/S; Advisory Board, AOP Orphan.

## Sources of funding

Supported in part by the David Johns Family Fund of the Cancer Research and Treatment Fund, New York, NY.
